# An Efficient Propagation Approach to Forcing Softwood Shoots from Epicormic Buds and Subsequent Rooting of *Paulownia elongata* S. Y. Hu

**DOI:** 10.1155/2024/1515489

**Published:** 2024-05-22

**Authors:** Muhammad Nadeem Maqbool, Faheem Aftab

**Affiliations:** Institute of Botany, University of the Punjab, Lahore, Pakistan

## Abstract

The current research describes the multiplication of *Paulownia elongata* S. Y. Hu, a timber plant, through the forcing of softwood shoots from epicormic buds under glasshouse conditions in spring and fall seasons. Different growth media were used to compare their effect on the forcing potential of epicormic buds. For this, 25–30-cm-long and 1.2–2-cm-diameter stem segments taken from the lower juvenile portion of a mother plant were placed horizontally in flat trays containing media, i.e., sterilized well-moistened sand, peat moss, perlite, and vermiculite individually. Furthermore, 4–6-cm-long forced softwood shoots were detached and treated with various concentrations of IBA (indole-3-butyric acid) and NAA (*α*-naphthyl acetic acid) either individually or in combinations for subsequent rooting. The response of shoot forcing was better in spring as compared to fall in terms of shoot length (cm), and number of shoots or leaves; however, an earlier bud break was observed during fall after 30 days of the initial experiment. The use of peat moss and vermiculite proved to be equally suitable for early bud break in both seasons, whereas in terms of shoot and leaf number as well as the shoot length (cm), the best outcome was observed in sand. Best rooting was observed at 3 gL^−1^ IBA + 3 gL^−1^ NAA in terms of root number per shoot, root length (cm), and days to root initiation while using sand as the growth medium after 50 days of the rooting experiment. The successfully established plantlets were further shifted to soil at Botanical Garden, University of the Punjab, Lahore, Pakistan, exhibiting an 87.5% survival rate. On the basis of the results obtained, it may be concluded that reasonable softwood shoot forcing in *P. elongata* may further be exploited for its mass scale nursery propagation as well as use in future in vitro studies.

## 1. Introduction

*Paulownia elongata* S. Y. Hu is a hardwood tree species. This exceptional genotype produces a lot of biomass, making it economically significant and a great source of feedstock for the biofuel sector [1, 2]. It controls soil and air pollution, facilitates land reclamation, and produces eye-catching flowers as an ornamental plant [3]. It is a deciduous tree, indigenous to China, and has since been introduced to Australia, Brazil, Europe, Japan, and the United States [4, 5]. In China, its growing habit ranges from temperate to nearly tropical zones [6].

The wood derived from *Paulownia* is valued for its physical toughness, light to medium fine-textured grain, and nice color as well as the fact that it is light and flexible but does not crack or distort easily [7]. Its wood is frequently used to make toys, furniture, plywood, aircrafts, and musical instruments [8]. As the demand for wood and wood products is increasing worldwide on a daily basis, it is crucial to meet the demand, especially in a country with a low forest land coupled with high pace of deforestation. In order to address the issues of demand for wood as well as CO_2_ emissions [9], it is necessary to look for prized tree species like *Paulownia elongata* with rapid growth and high CO_2_ absorption capacity.

*Paulownia* has already gained interest as short-rotation and potential bioenergy plant that can be used for both transportation fuel and carbon sequestration [10, 11]. Therefore, it is very important that this commercially significant plant needs to be domesticated outside of its natural habitats, such as in Pakistan where wood and energy demand is ever rising.

It is possible to reproduce *Paulownia* through seeds, but there are some limitations including seed dormancy and slower growth of seedlings than the plants developed through root or shoot cuttings or tissue culture [12]. In comparison with root cuttings, stem cuttings were considered quite challenging to grow [13]. Nonetheless, there are associated problems with growing root cuttings as well, such as physical injury to the root cortex and epidermis, deterioration from pathogen attack, and exposure to high temperatures [14]. It appears that none of these conventional propagation techniques are suitable for creating significant quantities of planting materials for the chosen elite trees [15, 16]. As a result, other vegetative propagation techniques such as softwood shoot forcing are essential to be worked out for *Paulownia's* clonal propagation.

A variety of commercially significant but recalcitrant woody plant species can be multiplied by forcing softwood shoots from epicormic buds of perennial plants [17]. In reality, latent (epicormic) buds are dormant meristematic tissues that were established during the juvenile stage of plant growth, which produce xylem and phloem during the favorable conditions to produce softwood sprouts [18]. Furthermore, it produces a relatively cleaner juvenile plant material with easier disinfection compared with explants obtained from field-grown plants. The word “cleaner” refers to the fact that in comparison with the plant shoots obtained from the field, the softwood shoots derived from the epicormic buds grown in a controlled environment of a glasshouse are relatively cleaner and, thus, better suited for further greenhouse or in vitro manipulation although they also need surface sterilization if the intended use of these softwood shoot-derived plant materials is, indeed, in vitro [19, 20]. This approach has been used to vegetatively propagate several woody plants successfully such as *Fraxinus pennsylvanica* Marsh. and *Acer saccharinum* L. [17], *Tectona grandis* L. [21], and *Gingko biloba* L. [22] for the sustainable availability of relatively healthy and cleaner explants for their in vitro establishment. On the other hand, there is a dearth of such research on *Paulownia elongata* S. Y. Hu that was chosen for this study as no published study on its softwood shoot forcing from the epicormic buds is currently accessible.

In this regard, forcing softwood shoots from epicormic buds was intended to be investigated as a novel method to produce young, disease-free *Paulownia elongata* plants as well as explant source materials by making use of various growth media (sand, peat moss, vermiculite, and perlite). Softwood shoots of *Paulownia elongata* as produced in this study can also be further used in in vitro research. Additionally, different IBA (indole-3-butyric acid) and NAA (*α*-naphthyl acetic acid) concentrations were employed to examine the impact on the rooting capacity of forced softwood shoots using different growth media. Successfully rooted plantlets were then acclimatized and established in the field conditions.

## 2. Materials and Methods

### 2.1. Softwood Shoot Forcing Using Stem Segments

For softwood shoot forcing from epicormic buds, large stem portions were removed from the lower juvenile parts of 3-year-old trees [*Paulownia elongata* S. Y. Hu, Taxon I.D # 200020795 (Quart J. Taiwan Mus. 12 : 41. 1959)] established at a local nursery at Model Town, Lahore. These trees were initially grown from seeds imported from China National Tree Seed Corporation, Jia 34 # Shenggunanli Chaoyang district: Beijing 100029, and the stock established in the growing area located some 15 km south-east of Lahore before their shifting to the retail nursery mentioned above. The stem portions were then cut into 36 sections, 25–30 cm long and 1.2–2 cm in diameter, three each to be placed horizontally, half embedded in a well-moistened specific medium prefilled in an iron-alloy tray (55 × 32 × 6.6 cm). Twelve flat trays were used, three trays (three replicates) for each of four growth media (sand, peat moss, perlite, and vermiculite) with 3 logs per replicate. The four tested media in this study were local commercially available coarse sand (bulk density 1.6 g·cm^−3^, total porosity 35%, and pH 5.5–6.5), peat moss (Nord Agri Ltd. Elizabetes iela, Riga, LV1001, Latvia, bulk density 0.086 g·cm^−3^, total porosity 80%, and pH 5.0–5.6), perlite (Gulf Perlite Dubai, UAE, bulk density 0.1–1.1 g·cm^−3^, total porosity 70–85%, and pH 6–8.5), and vermiculite (Gulf Vermiculite Dubai, UAE, bulk density 0.07–0.1 g·cm^−3^, total porosity 80–85%, and pH 7–7.5), thus making a total of 12 trays with each medium replicating thrice and placed under glasshouse conditions mentioned below.

Two sets of softwood shoot forcing experiment were performed in the third week of February 2020, with an average temperature of 18°C and 55% relative humidity (RH) as well as in the last week of September 2020, with an average temperature of 36°C and 70% RH. A 16-h photoperiod and a light intensity of 200–250 *μ*mol·m^−2^·s^−1^ were maintained. The same experiment was repeated in mid-February 2021 with 17°C average temperature and 60% RH and in the end of September, 2021 with an average temperature of 39°C and 75% RH. The global positioning system coordinates (GPSs) of the experimental site were 31°30′04.4″N and 74°18′27.7″E. Regular watering to these stem sections was done manually with 650 ml water per tray per day with the help of spray bottles. On alternate days, 0.18% H_2_O_2_ solution was sprayed in order to protect stem sections from microbial or fungal growth by following the method of Aftab et al. [17]. Data were tabulated for days to bud break, shoot number, number of leaves per shoot, and length (cm) of softwood shoots after 30th day of the initial experiment.

### 2.2. Rooting Potential of Harvested Soft Wood Shoots

After 30 days of the experiment's initiation, epicormic softwood shoots of 4–6 cm in length were removed from the stem sections with some basal tissue for the rooting response. These shoots were taken during April 2020 and March 2021. Basal ends of the excised shoots were placed in distilled water instantly after cutting to avoid drying, and the larger leaves were cut into half transversally to reduce transpiration. The treatment of the basal parts of forced softwood shoots was done by dipping them for 40 seconds in either 1, 2, or 3 gL^−1^ solution of NAA (Sigma-Aldrich®)] or IBA (Sigma-Aldrich®) alone or in combination. The treated shoots were placed vertically to an anchorage of 2–2.5 cm in 9-cm-deep and 11.5-cm-wide plastic pots filled up with little more than three-fourth sterilized sand. These plastic pots were covered with perforated polythene bags of appropriate size to maintain humidity, which were then placed on racks equipped with cool white florescent 40-watts tube lights (Philips Pakistan Ltd. Karachi) at 25 ± 2°C under 16-h photoperiod (32 *μ*mol·m^−2^·s^−1^).

Foliar water spray on these shoots was also carried out every day, while 0.18% H_2_O_2_ (v: v) solution was sprayed on alternate days. Data were collected for days to root induction, root number, and root length (cm) after 50 days of rooting experiment initiation.

### 2.3. Acclimatization

Rooted softwood shoots were then shifted to earthen pots (14 cm wide and 11 cm deep) each filled with course sand, garden soil, and peat moss (2 : 1 : 1; v/v) for 5 weeks to establish them further in the glasshouse. The moisture content of the earthen pots was maintained by regular watering as well as by covering the pots initially (for 3 weeks) with polythene bags of appropriate size. After 5 weeks, the plants in pots were shifted to exposed nursery conditions (outside the glasshouse) for another 2 weeks. The successfully established plants were further transplanted in soil at Botanical Garden, University of the Punjab, Lahore. Transplantation survival was noted.

### 2.4. Experimental Design and Data Analysis

For this study, a completely randomized design was adopted. Softwood shoots were forced from epicormic buds with three replicates of four growth media during spring and fall 2020 followed by its subsequent run in 2021. Similarly, two independent trials were conducted for the rooting potential of softwood shoots in pots using nine auxins treatments each with three replicates (pots) with one shoot per pot in two independent trials. The data were subjected to the analysis of variance (ANOVA) and *t*-test (*p*=0.05) using SPSS Release 21.0. PCA for interactive correlation between variables was done using OriginPro® 2024.

## 3. Results and Discussion

### 3.1. Softwood Shoot Forcing Using Stem Segments

**Figure 1:**
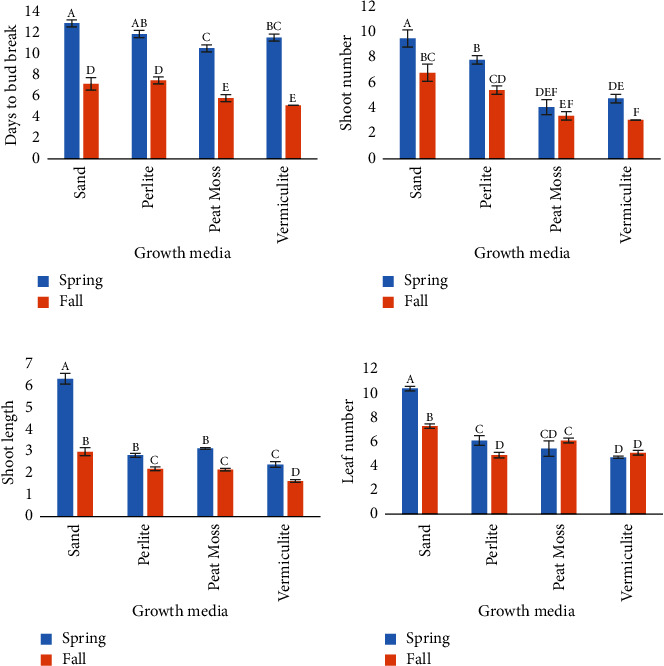
Effect of sand, perlite, peat moss and vermiculite on various growth parameters of softwood shoots forced from epicormic buds in *Paulownia elongata* S. Y. Hu. (a)-Days to bud break, (b)-shoot number, (c), shoot length (cm) and (d)-leaf number values shown are means (± SE). Variation in alphabets represents statistical difference among growth media (*p* = 0.05).

**Figure 2:**
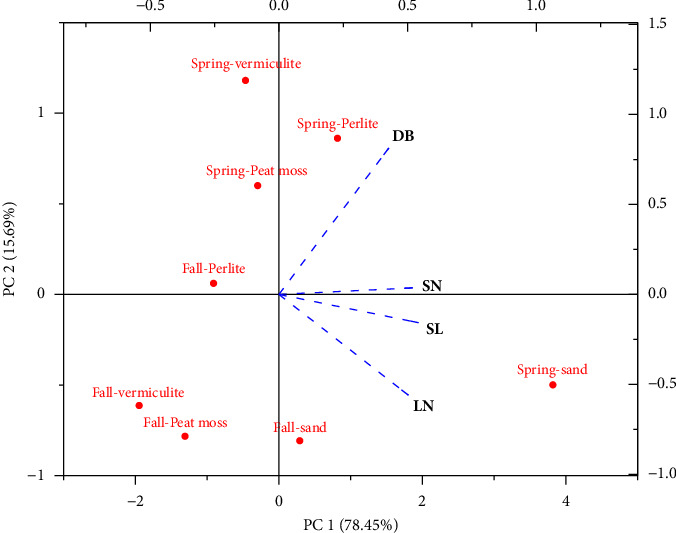
Principal component analysis (PCA) biplots presenting the interactive correlation of different growth media along with two seasons (spring and fall) on measured growth parameters (days to bud break: DB; shoot number: SN; shoot length (cm): SL; leaf number: LN).

**Figure 3:**
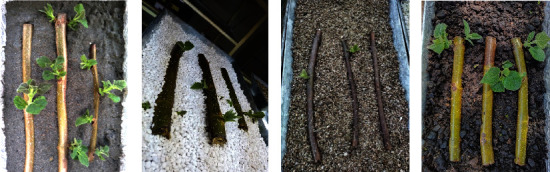
Forcing of softwood shoots from epicormic buds using different growth media in *Paulownia elongata* during spring season after 30 days of initial experiment. (a) Forced softwood shoots in sand. (b) Large stem segments showing the emergence of softwood shoots in perlite. (c) Browning of stem segments and less number of shoots in vermiculite. (d) Minimum shoot number in peat moss. Bar = 2 cm.

**Figure 4:**
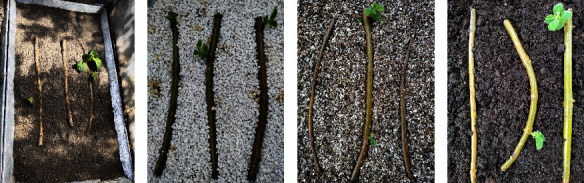
Forcing of softwood shoots from epicormic buds using different growth media in *Paulownia elongata* during the fall season after 30 days of the initial experiment. Overall, growth media: (a) sand, (b) perlite, (c) vermiculite, and (d) peat moss, gave lesser response during fall time experiment as compared to spring in terms of shoot number, shoot length, and number of leaves. Bar = 4 cm.

Results on softwood shoot forcing were quite comparable in both yearly runs of the experiments, i.e., the one conducted in the spring and fall of the year 2020 and the other during the same time in 2021. In both seasons, softwood shoot forcing was successful; however, an experiment conducted in spring (February) resulted in better growth parameters in terms of shoot length (cm), shoot, and leaf numbers (Figures 1–4). Similar to our findings with *Paulownia elongata*, Preece et al. [23] had observed earlier that the optimal time for softwood shoot forcing using stem portions of various plant species, e.g., oak-leaf hydrangea, common lilac, and sugar maple, is from midwinter until the end of spring, depending on the kind of woody plants. However, forcing of softwood shoots is also possible in some plant species until fall. It is probably due to the dormancy of epicormic buds prior to the onset of the winter season in response to environmental stimuli [24]. In plants, the control of dormancy is believed to be because of genetic, physiological, environmental, and hormonal factors [25, 26]. After passing through the chilling temperature, normally dormancy is released as the cell cycle, hormonal activity, and starch degradation into soluble sugars begin [27, 28]. It is believed that woody plant species of temperate regions have to undergo the dormant stage during the winter season to survive under unfavorable conditions and then start to grow again in spring under suitable temperature and humidity [29].

During the spring time experiments, considering overall performance, sand proved to be the most favorable growth substrate for softwood shoot forcing using stem sections as compared to perlite, peat moss, and vermiculite in the current study (Figure 1). Minimum days to bud break (10.33) were noted in peat moss, while maximum days (12.67) were in sand (Figure 1(a)). Softwood shoot forcing in peat moss was significantly different (*p*=0.05) from sand, but no statistical difference was observed with vermiculite and perlite. A higher number of shoots were observed in sand (9.33) whereas the least (4.00) in peat moss (Figure 1(b)). The number of softwood shoots in sand was significantly different from peat moss, perlite, and vermiculite in which perlite was also significantly different from peat moss and vermiculite. According to Pramanik et al. [30], cocopeat supported the highest growth of plantlets in external conditions as compared to perlite, vermiculite, and sand in *Bacopa monnieri* L. Panigrahi et al. [31], on the other hand, found the mixture of vermiculite, sand, and organic matter (1 : 1 : 1 v/v) as the best growth substrate for plants instead of using them alone for *Chlorophytum borivilianum* Santapau & R. R. Fern. Best growth parameters were noted under natural habitat using garden soil, sand, and farmyard manure (2 : 1 : 1; v/v) in *Gloriosa superba* L. [32]. Gantait et al. [33] proved course sand to be the most suitable growth medium for the optimal multiplication of *Bambusa balcooa* Roxb through culm cuttings in comparison with other three media (soil, vermiculite and 1 : 1 (v/v) soil + sand). In our study, the longest softwood shoots (6.23 cm) were observed in sand while the smallest (2.35 cm) in vermiculite (Figure 1(c)). Data with respect to the shoot length in sand have also shown a statistical difference from all other growth media used. Vermiculite had the least efficient effect on softwood shoot forcing exhibiting rotting or necrosis of the leaves probably due to the deficiency of O_2_ by excessive water retention [34]. Similarly, a maximum leaf number (10.23) was observed in sand whereas the minimum (4.63) in vermiculite (Figure 1(d)). A significant difference was observed in sand in terms of number of leaves from vermiculite, perlite, and peat moss. Similar findings were observed by Noreen and Aftab [22] for *Ginkgo biloba*. They also observed a maximum number of leaves and a maximum shoot length in sand while a minimum in vermiculite.

The experiment setup in fall has shown a similar response but with a lesser number of softwood shoots and leaves and a decrease in the shoot length (Figures 1(b)–1(d)). However, an early response to bud break was noted during fall as shown in Figure 1(a). Moreover, similar to the springtime experiment, sand was found to be a better medium supporting a better shoot length, and shoot and leaf number during fall as well, except for the bud break response, which was earliest in the case of vermiculite (Figure 4).

### 3.2. Principal Component Analysis (PCA)

The interaction of the two seasons (spring and fall) and different growth media along their correlation with the variables is displayed in a principal component analysis (PCA)-based biplot (Figure 2). The combined biplot displayed 94.14% of the total variation, with PC1 contributing the maximum variation (78.45%) and PC2 showing 15.69%. The parameters presented in the same quadrate are nonsignificantly different. Among different growth media, sand was noted as the significant medium in both seasons presented in the same quadrate and showed a positive correlation with SN, SL, and LN. Peat moss and vermiculite showed negative correlation with observed parameters. Perlite gave positive correlation with DB. Overall, the spring season resulted in better than fall for most of the parameters. Therefore, this technique can be employed to propagate *Paulownia elongata* as multipurpose tree round the year even during the fall season to fulfill the demand for high-quality wood along with other benefits such as a source of solid biofuel, role in pharmaceutical industry, and ornamental aspect in regions where this plant is exotic.

### 3.3. Rooting Potential of Harvested Softwood Shoots

**Figure 5:**
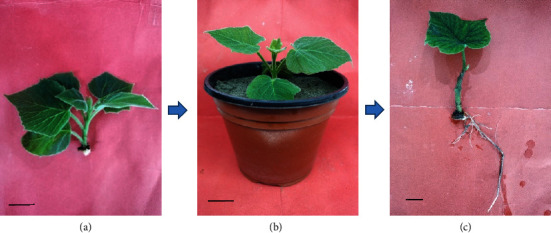
Root formation of forced softwood shoots in *Paulownia elongata* on sand using auxins (NAA and IBA at 3 gL^−1^ each) under controlled culture conditions. (a) Excised epicormic softwood shoot of the stem section with little basal tissue. (b) Softwood shoot planted in plastic pot containing sand after treating with auxin. (c) Plantlet showing extensive rooting response after 50 days of transplantation. Bar = 2 cm.

Sand was found to support quite reasonable rooting of softwood shoots in 50 days (Figure 5) compared with peat moss, perlite, or vermiculite used either individually or in combinations (data not shown). As Karrenberg et al. [35] reported that the characteristics of the growth substrates being employed were the key determinants of root anchoring, this might have been the case with sand in our study that proved to be a good substrate to support rooting in *Paulownia*. Sand was also found to be the best anchoring medium in a similar work by Akram and Aftab [21] and Noreen and Aftab [22] on Teak and *Ginkgo*, respectively. Gaintait et al. [33] also concluded that the sand gave the quickest root initiation presumably due to high aeration characteristics than vermiculite. The porosity of 35%, ≤2 mm particle size, enough moisture holding capacity and a sufficient oxygen diffusion rate are the characteristics of sand [36, 37]. Sand may have been effective because of these characteristics both for forcing softwood shoots and for later root induction in *Paulownia*. Furthermore, sand provides a sufficiently permeable environment for root establishment without suffering injury as reported for *Azadirachta indica *A. Juss [38]. Similarly, studies of Pill and Goldberger [39] indicated that sand was an efficient rooting medium as compared to other media such as perlite. Perlite has got a larger particle size and 70–85% of total porosity, which may not furnish sufficient anchorage to little harvested forced softwood shoots.

Our findings with regard to rooting were also in line with those of Sardoei [40] who reported significant rooting in sand as compared to perlite in *Psidium guajava* L. However, Stuepp et al. [41] have also recommended vermiculite and carbonized rice hulls (1 : 1) for efficient root induction in mini-cuttings of *Paulownia fortunei* var. Mikado stumps.

**Figure 6:**
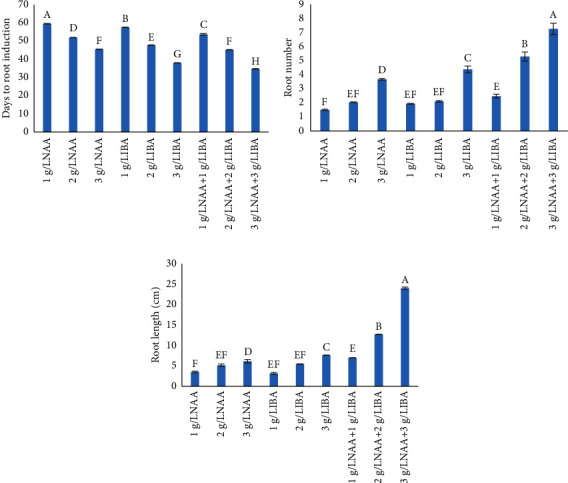
Effect of auxin (NAA and IBA) treatments on various parameters of rooting of forced softwood shoots using sand as the growth medium in *Paulownia elongata*. (a) Days to root initiation, (b) root number, and (c) root length (cm). Values are mean (±SE), and the variation in alphabets shows the statistical difference between different concentrations of hormones (*p*=0.05).

In this study, individual treatments of either NAA or IBA (1 gL^−1^ each) were least effective in terms of root length, root number, or days to root initiation. Increasing concentrations of NAA and IBA from 1 to 3 gL^−1^ supported better rooting, but it was with both hormones in combination that supported the most efficient rooting. Earliest root initiation (45.22 days) was recorded by using 3 gL^−1^ NAA, while a delayed response (59.13 days) was exhibited at 1 gL^−1^ NAA. Higher concentration of NAA (3 gL^−1^) proved to be more effective with respect to the root number (3.66 ± 0.06) and root length (6.07 ± 0.43 cm). Using IBA, the results were also in line with NAA. A delayed response to root initiation (57.18 ± 0.09 days) was observed at 1 gL^−1^ IBA, while an early rooting response (37.73 ± 0.08 days) was observed at its 3 gL^−1^ level. Similarly, the maximum root number (4.36 ± 0.24) and length (7.55 ± 0.03 cm) were also observed at 3 gL^−1^ IBA as shown in Figure 6. The results indicated that IBA was more effective for root development than NAA when used at the same concentrations. Similar results regarding the role of IBA on adventitious root induction of *Gingko biloba* were recorded by Pandey et al. [42]. In fact, Zayova et al. [43] also observed that IBA was more effective for root development than NAA and IAA with the best results at 0.5 mgL^−1^ IBA using ½ MS (Murashige and Skoog) medium [44]. Noreen and Aftab [22] reported that a rooting response in the softwood shoots derived from epicormic buds of *Ginkgo biloba* was best on sand after IBA treatment. Shepherd et al. [45] worked on the rooting of cuttings obtained from epicormic shoots of tea tree (*Melaleuca alternifolia* (Maiden & Betche) Cheel) under a variety of propagation circumstances using IBA. Muhamad et al. [12] also concluded that IBA was more productive than NAA for in vitro rooting in *Paulownia* spp. when supplemented with MS.

NAA and IBA in combination (3 gL^−1^ each) resulted in best rooting in terms of maximum number of the longest roots per plantlet (Figure 6). Earliest root initiation (34.45 days), maximum number (7.23 ± 0.40), and longest roots (23.78 ± 0.27 cm) were obtained at 3 gL^−1^ each of NAA and IBA in combination. We support the findings of Filipova et al. [46] who reported the combination of 0.5 mgL^−1^ NAA and 4 mgL^−1^ IBA for an efficient root development system in *Paulownia*. Our reported concentrations for the two growth regulators though different do indicate that a range of levels of NAA and IBA may work for the rooting of this important tree species. Different combinations of IBA, IAA, and NAA have also been reported to be superior for the rooting of stem cuttings of *Ginkgo* [42, 47]. As the activation of polysaccharide hydrolysis is affected by auxins to release physiologically active sugars and, therefore, energy for root induction-related meristematic activity [48], the combined effect of IBA and NAA as reported in this study might have triggered better polysaccharide hydrolysis, resulting in an efficient rooting in *Paulownia*.

### 3.4. Acclimatization of Rooted Plantlets

**Figure 7:**
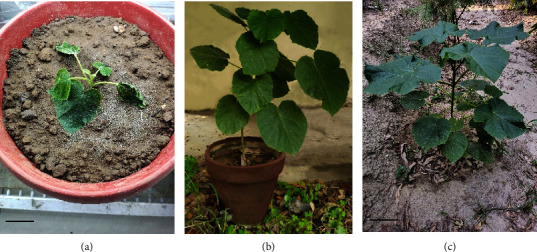
Successful acclimatization of rooted plantlets. (a) Rooted plantlet survived after 10 days of transplantation. Bar = 2 cm. (b) Earthen pot showing the vigorous growth of the rooted plantlet after 40 days of initial shifting from the culture room to the glasshouse. Bar = 2 cm. (c) 3-month-old plant established in the field at Botanical Garden, University of the Punjab, Lahore, Pakistan. Bar = 10 cm.

As sand proved to be the best growth medium for rooting response, hardening of 40 rooted plantlets was initiated using sand enhanced with soil and peat moss (2 : 1 : 1 v/v) for further growth and establishment in the field (Figure 7). Transplantation survival percentage was 87.5. A key component of a successful acclimatization was maintaining the humidity quotient, which was ensured by water spraying and subsequent covering the potted plantlets with polythene bags [49, 50]. According to Gantait et al. [33], using sand, farmyard manure, and cocopeat (1 : 1 : 1 v/v/v) increased the rate of survival of fresh roots in *Bambusa balcooa* by 100%. Cocopeat was found by Pramanik et al. [30] to be the best substrate for *Bacopa monnieri*, with a 100% survival rate. In their investigation into the acclimatization protocol for *Paulownia* species, Muhamad et al. [12] discovered that the best growth substrate mixture with 100% survivability was sand and peat moss (1 : 2 v/v). Akramian et al. [51] reported cocopeat and perlite (2 : 1 v/v) as the best horticulture substrate with 100% survivability while studying *Philodendron* cv. Birkin.

## 4. Conclusion

The successful forcing of softwood shoots of *Paulownia elongata* and their subsequent rooting is a step towards future exploitation of this as-yet-unexploited technique for growing this important tree species outside of its natural habitat. We recommend using sand as the growth medium and the combination of IBA and NAA (3 gL^−1^ each) for their rooting. As sand is readily available and considerably less expensive than other growth media, it may also fulfill the requirement of cost-effectiveness, a major consideration in the mass scale propagation of ornamental or economically important plants. Spring in both years is proved to be the best time to force softwood shoots probably due to suitable mild temperature and relative humidity.

The findings of the current investigation highlight the fact that the propagation of *Paulownia elongata* through softwood shoot forcing and its subsequent root induction holds good promise for the mass propagation of this plant species. In addition, this method can be employed to obtain a relatively cleaner plant material and suitable explants for the use in various aspects of in vitro research.

The following suggestions for future research may be considered:

- Relatively cleaner and juvenile explant source for in vitro studies.
- It can be employed for the propagation of *Paulownia* under nursery conditions and then used for further studies.
- As this elite plant is known for precious wood, large-scale production can be achieved through this efficient technique.
- Softwood shoot forcing experiment can be compared under controlled culture conditions and a natural environment.
- Diameter of the stem segments can be compared for shoot vigor.

## Data Availability

The relevant raw data shall be provided by the corresponding author upon request.
